# Antimicrobial susceptibility pattern of bacterial isolates from wound infection and their sensitivity to alternative topical agents at Jimma University Specialized Hospital, South-West Ethiopia

**DOI:** 10.1186/1476-0711-13-14

**Published:** 2014-04-14

**Authors:** Mohammedaman Mama, Alemseged Abdissa, Tsegaye Sewunet

**Affiliations:** 1 Department of Medical Laboratory Science, Arba Minch University, Arba Minch, Ethiopia; 2 Department of Medical Laboratory Science and Pathology, Jimma University, Jimma, Ethiopia

**Keywords:** Bacterial pathogens, Drug resistance, Wound infection, Jimma, Ethiopia

## Abstract

**Background:**

Wound infection is one of the health problems that are caused and aggravated by the invasion of pathogenic organisms. Information on local pathogens and sensitivity to antimicrobial agents, and topical agents like acetic acid is crucial for successful treatment of wounds.

**Objectives:**

To determine antimicrobial susceptibility pattern of bacterial isolates from wound infection and their sensitivity to alternative topical agents at Jimma University Specialized Hospital.

**Methods:**

A cross sectional study was conducted among patients with wound infection visiting Jimma University Specialized Hospital, from May to September 2013. Wound swab was collected using sterile cotton swabs and processed for bacterial isolation and susceptibility testing to antimicrobial agents, acetic acid, hydrogen peroxide and dabkin solution following standard bacteriological techniques. Biochemical tests were done to identify the species of the organisms. Sensitivity testing was done using Kirby- Baur disk diffusion method. Minimum inhibitory and bactericidal concentration was done using tube dilution method.

**Results:**

In this study 145 bacterial isolates were recovered from 150 specimens showing an isolation rate of 87.3%. The predominant bacteria isolated from the infected wounds were Staphylococcus aureus 47 (32.4%) followed by Escherichia coli 29 (20%), Proteus species 23 (16%), Coagulase negative Staphylococci 21 (14.5%), Klebsiella pneumoniae 14 (10%) and Pseudomonas aeruginosa 11 (8%). All isolates showed high frequency of resistance to ampicillin, penicillin, cephalothin and tetracycline. The overall multiple drug resistance patterns were found to be 85%. Acetic acid (0.5%), Dabkin solution (1%) and 3% hydrogen peroxide were bactericidal to all isolated bacteria and lethal effect observed when applied for 10 minutes.

**Conclusions:**

On in vitro sensitivity testing, ampicillin, penicillin, cephalothin and tetracycline were the least effective. Gentamicin, norfloxacin, ciprofloxacin, vancomycin and amikacin were the most effective antibiotics. Acetic acid (0.5%), dabkin solution (1%) and H_2_O_2_ (3%) were bactericidal to all isolates.

## Introduction

The primary function of intact skin is to control microbial populations that live on the skin surface and to prevent underlying tissue from becoming colonized and invaded by potential pathogens [1].

Exposure of subcutaneous tissue following a loss of skin integrity (i.e. wound) provides a moist, warm, and nutritious environment that is conducive to microbial colonization and proliferation. Since wound colonization is most frequently poly-microbial, involving numerous microorganisms that are potentially pathogenic, any wound is at some risk of becoming infected [2].

Infection in wound constitutes a major barrier to healing and can have an adverse impact on the patient’s quality of life as well as on the healing rate of the wound. Infected wounds are likely to be more painful, hypersensitive and odorous, resulting in increased discomfort and inconvenience for the patient [3].

The prevalent organisms that have been associated with wound infection include *Staphylococcus aureus (S. aureus)* which from various studies have been found to account for 20-40% and *Pseudomonas aeruginosa (P. aeruginosa)* 5-15% of the nosocomial infection, with infection mainly following surgery and burns. Other pathogens such as Enterococci and members of the Enterobacteriaceae have been implicated, especially in immune compromised patients and following abdominal surgery [4].

Wound healing needs a good healthy environment so that the normal physiological process will result in a normal healing process with minimal scar formation. One of the most important strategies to keep the process of healing ongoing is to sterilize damaged tissue from any microbial infection [5].

Continued use of systemic and topical antimicrobial agents has provided the selective pressure that has led to the emergence of antibiotic resistant strains which in turn, has driven the continued search for new agents. Unfortunately, the increased costs of searching for effective antimicrobial agents and the decreased rate of new drug discovery has made the situation increasingly worrisome [6].

Hence the present study is designed to update profile of bacteria present in wounds, their sensitivity to antibiotics and sensitivity to alternative topical agents at Jimma University Specialized Hospital, Jimma, Ethiopia.

## Methods

### Study design and area

A cross sectional study was conducted at Jimma University specialized hospital (JUSH), which is located 354 Km away from Addis Ababa, South West, Ethiopia, from May to September 2013. JUSH is a referral hospital in southwestern part of the country.

### Sampling procedure

A questionnaire was used to obtain data from the patient after obtaining an informed consent from the patient/guardians. Open wound swabs were aseptically obtained after the wound immediate surface exudates and contaminants were cleansed off with moistened sterile gauze and sterile normal saline solution. Dressed wounds were cleansed with sterile normal saline after removing the dressing. The specimen was collected on sterile cotton swab by rotating with sufficient pressure. Double wound swabs were taken from each wound at a point in time to reduce the chance of contamination. The samples were transported to the laboratory after collection using Amies transport media.

### Culture and identification

Swabs collected were streaked on blood agar and MaCconkey agar (oxoid) by sterile inoculation loop. The plates were incubated at 35–37°C for 24–48 hours. Preliminary identification of bacteria was based on colony characteristics of the organisms. Such as haemolysis on blood agar, changes in physical appearance in differential media and enzyme activities of the organisms. Biochemical tests were performed on colonies from primary cultures for identification of the isolates. Gram-negative rods were identified by performing a series of biochemical tests (oxoid). Namely: Kliger Iron Agar (KIA), Indole, Simon’s citrate agar, Lysine Iron Agar (LIA), urea and motility. Gram-positive cocci were identified based on their gram reaction, catalase and coagulase test results.

### Antibacterial susceptibility testing (AST)

Susceptibility testing was performed by Kirby-Bauer disk diffusion technique according to criteria set by CLSI 2011. The inoculum was prepared by picking parts of similar test organisms with a sterile wire loop and suspended in sterile normal saline. The density of suspension to be inoculated was determined by comparison with opacity standard on McFarland 0.5 Barium sulphate solution. The test organism was uniformly seeded over the Mueller-Hinton agar (oxoid) surface and exposed to a concentration gradient of antibiotic diffusing from antibiotic-impregnated paper disk into the agar medium, and then incubated at 37°C for 16–18 hours. Diameters of the zone of inhibition around the discs were measured to the nearest millimeter using a ruler and classified as sensitive, intermediate, and resistant according to the standardized table supplied by CLSI 2011.

The drugs tested for both gram negative and gram positive bacteria were ampicillin (10 μg), ciprofloxacin (5 μg), norfloxacin (10 μg), cephalothin (30 μg), gentamicin (10 μg), tetracycline (30 μg), cotrimoxazole (25 μg), chloramphenicol (30 μg), doxycycline (30 μg), naldixic acid (15 μg) and ceftriaxone (30 μg). Penicillin G (10 IU), erythromycin (15 μg) and vancomycin (30 μg) were used for only gram positive bacterial isolates (oxoid). These antimicrobial selected based on the availability and prescription frequency of these drugs in the study area.

### Minimum inhibitory and bactericidal concentration determination

Freshly prepared solutions of different concentrations 0.5%, 1%, 1.25%, 1.5%, 1.75% and 2% of acetic acid was prepared by adding in to 100 ml of sterile distilled water, while (0.025%, 0.25%, 0.5%, 1%, 1.25%, 1.5%, 1.75% and 2% concentration of dabkin solution were made by adding in to 100 ml of sterile distilled water. Hydrogen peroxide (3%) was used. The minimum inhibitory concentrations (MICs) and minimum bactericidal concentrations (MBCs) of the antimicrobial agents were determined for each isolate by tube dilution method then sub cultured on agar plate. This technique was done by mixing sterile 4.9 ml of tryptone soya broth (oxoid) with 5 ml of each serially obtained concentration of antimicrobial agents. The test organisms from growth on nutrient agar plates incubated at 37°C for 18 hrs were suspended in sterile saline solution and adjusted to match a turbidity of 0.5 McFarland standards. To get a final volume of 10 ml, 0.1 ml of standardized bacterial suspension was inoculated in each tube. After overnight incubation aerobically at 36-37°C the tubes were examined macroscopically for visible evidence of bacterial growth in the form of turbidity by comparing with the control tubes. Two control tubes were employed; one was a row of positive control tubes containing only the nutrient broth and each of the microorganisms, while negative controls were set up as follows: nutrient broth only; nutrient broth and sterile antimicrobial agents. MIC was recorded as the lowest concentration of dabkin solution or acetic acid that inhibited bacterial growth (no visible growth or turbidity). The minimum bactericidal concentration were determined from the test tubes used in the determination of MIC, the tubes that showed no visible growth were sub cultured onto freshly prepared nutrient agar at 37°C for 48 hours. Plates were examined and MBC was recorded as the lowest concentration of dabkin solution and acetic acid at which no colony was formed on the plate.

### Data analysis

Data was edited, cleaned, entered and analyzed using statistical package for social science (SPSS) version 17. Descriptive analysis such as frequencies and mean were used. The chi-square test was employed to compare the association of socio-demographic data, wound type, location with wound infection status of the patients. P-value of < 0.05 was considered to indicate statistically significant differences. The result was presented using tables and charts.

### Ethics

Ethical clearance was obtained from the ethical committee of Jimma University college of Public Health and Medical Science. Written informed consent was obtained from all study participants.

## Results

A total of 150 specimens were collected from patients with clinical evidence of wound infection (patients with complaints of discharge, pain, swelling, foul smelling and chronic wound) from May to September, 2013. The subjects included 107 (71.3%) males and 43 (28.7%) females. The ages of the patients ranged from 6 months to 90 years with mean age of 31.68 ± 17.12 (Table 1).

**Table 1:** Wound infection and socio-demographic characteristics of the patients at JUSH, Jimma, May-September, 2013

**Demographic characters**	**Infected No. (%)**	**Not infected No. (%)**	**Total No. (%)**
**Sex**			
Male	96 (89.7)	11 (10.3)	107 (71.3)
Female	35 (81.4)	8 (18.6)	43 (28.7)
**Total**	**131 (87.3)**	**19 (12.7)**	**150 (100)**
**Age in years**			
≤ 15	21(87.5)	3 (12.5)	24 (16)
16-30	54 (87.1)	8 (13)	62 (41.3)
31-44	25 (86.2)	4 (13.8)	29 (19.3)
45-59	17 (89.5)	2 (10.5)	19 (12.7)
≥ 60	14 (87.5)	2 (12.5)	16 (10.7)
**Total**	**131 (87.3)**	**19 (12.7)**	**150 (100)**

Forty five (30%) samples screened was obtained from the leg, while 22 (14.7%) of the wound affected the abdomen. Sixty five of the cases (43.3%) seen were trauma, followed by 34 (22.7%) which were postoperative wound (Table 2).

**Table 2:** Wound type and location from patients with infected wounds at JUSH, Jimma, May-September 2013

**Wound location**	**Number (%)**
Leg	45 (30.0)
Abdomen	22 (14.7)
Hand	13 (8.7)
Buttocks	13 (8.7)
Foot	13 (8.7)
Head and neck	12 (8.0)
Back	11 (7.3)
Genitals	8 (5.3)
Breast and chest	7 (4.7)
Armpit	3 (2.0)
Others	3 (2.0)
**Total**	**150 (100.0)**
**Type of wound**	
Trauma	65 (43.3)
Postoperative wound	34 (22.7)
Abcess	31 (20.7)
Ulcers	10 (6.7)
Burn wound	5 (3.3)
Diabetic foot ulcers	5 (3.3)
**Total**	**150 (100.0)**

### Bacterial profile

**Figure 1:**
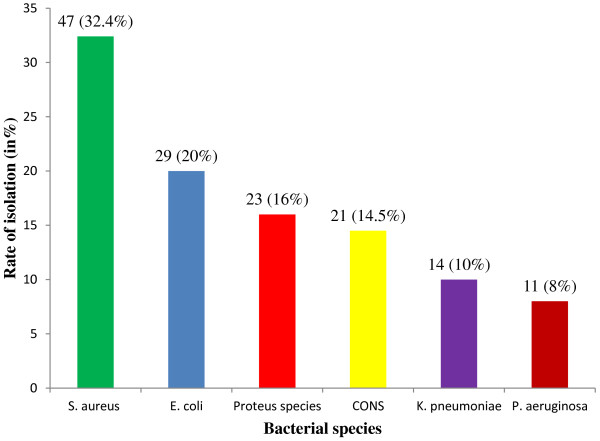
Percentage of bacteria isolated from patients with infected wounds at JUSH, Jimma, May-September, 2013.

Of the 150 swabs 131 (87.4%) were culture positive for bacterial pathogens, while 19 (12.6%) were bacteriologically sterile. The presence of only one species isolated from each sample was the most frequent (91.6%) while, more than one species were isolated from (8.4%) of the total swabs. A total of 145 bacterial isolates were obtained, 77 (53%) were gram negative while 68 (47%) were gram positive. *S. aureus* was the predominant organism isolated 47 (32.4%), followed by *Escherichia coli (E. coli)* 29 (20%), *Proteus* spps 23 (16%), coagulase negative *Staphylococci* 21 (14.5%), *Klebsiella pneumoniae (K. pneumoniae)* 14 (10%) and *P. aeruginosa* 11 (8%) (Figure 1)*.*

### Antimicrobial susceptibility pattern of bacterial isolates

#### Gram positive bacteria

Gram positive bacteria were tested against selected 14 antibiotics. The results obtained showed that the organisms varied in their susceptibility to all the antimicrobials used. Majority of them showed multi-resistances (resistance to two or more classes of antimicrobials). Rate of isolates resistant to ampicillin was 94%, followed by penicillin G, 86.8%. All isolates were 100% susceptible to vancomycin and amikacin, and showed low resistance to norfloxacin (10%), ciprofloxacin (10%), sulphamethoxazole trimethoprim (8.8%) and gentamicin (8.8%) (Table 3).

**Table 3:** Antibiotic susceptibility pattern of gram positive bacteria isolated from patients at JUSH, Jimma, May-September 2013

**Isolates**	**RXN**	**Antimicrobial agents (%)**													
		**CN**	**VA**	**AK**	**E**	**C**	**SXT**	**NOR**	**P**	**KF**	**CRO**	**TE**	**CIP**	**AP**	**DO**
*S.aures* (n = 47)	S	45 (96)	47 (100)	47 (100)	40 (85.1)	40 (85.1)	44 (94)	45 (96)	4 (8.5)	33 (70.2)	40 (85.1)	23 (49)	45 (96)	2 (4.3)	34 (72.4)
	R	2 (4)	-	-	7 (14.9)	7 (14.9)	3 (6)	2 (4)	43 (91.5)	14 (29.8)	7 (14.9)	24 (51)	2 (4)	45 (95.7)	13 (27.6)
CONS (n = 21)	S	17 (81)	21 (100)	21 (100)	13 (62)	14 (67)	18 (86)	16 (76.2)	5 (24)	6 (29)	15 (71.4)	10 (48)	16 (76.2)	2 (9.5)	15 (71.4)
	R	4 (19)	-	-	8 (38)	7 (33)	3 (14)	5 (23.8)	16 (76)	15 (71)	6 (28.6)	11 (52)	5 (23.8)	19 (90.5)	6 (28.6)
**Total (n = 68)**	**S**	**62 (91.2)**	**68 (100)**	**68 (100)**	**53 (78)**	**54 (79.4)**	**62 (91.2)**	**61 (90)**	**9 (13.2)**	**39 (57.4)**	**55 (81)**	**33 (48.5)**	**61 (90)**	**4 (6)**	**49 (72.1)**
	**R**	**6 (8.8)**	**-**	**-**	**15 (22)**	**14 (20.6)**	**6 (8.8)**	**7 (10)**	**59 (86.8)**	**29 (42.6)**	**13 (19)**	**35 (51.5)**	**7 (10)**	**64 (94)**	**19 (27.9)**

KEY: S: Sensitive; R: Resistant; −: zero; CN: Gentamicin; V: Vancomycin; AK: Amikacin; E: Erythromycin; C: Chloramphenicol; SXT: Trimethoprim-sulphamethoxazole; NOR: Norfloxacin; P: Penicillin; KF: Cephalothin; CRO: ceftriaxone; TE: Tetracycline; CIP: Ciprofloxacin AP: Ampicillin; DO: Doxycycline.

#### Gram negative bacteria

The susceptibility patterns of gram negative bacteria (n = 77) isolated from wound infections and tested against selected 11 antimicrobial agents. Rate of isolates resistant to ampicillin was 96%, followed by cephalothin, 92.4% (Table 4).

**Table 4:** Antibiotic susceptibility pattern of gram negative bacteria isolated from patients at JUSH, Jimma, May-September 2013

**Isolates**	**RXN**	**Antimicrobial agents (%)**										
		**CN**	**C**	**SXT**	**NA**	**NOR**	**KF**	**CRO**	**TE**	**CIP**	**AP**	**DO**
*E.coli* (n = 29)	**S**	14 (48.3)	10 (34.5)	13 (45)	17 (59)	16 (55.2)	0	11 (38)	6 (21)	19 (66)	-	16 (55.2)
	**R**	15 (51.7)	19 (65.5)	16 (55)	12 (41)	13 (44.8)	29 (100)	18 (62)	23 (79)	10 (34)	29 (100)	13 (44.8)
*Proteus Spp* (n = 23)	**S**	17 (74)	16 (70)	14 (61)	15 (65.2)	20 (87)	3 (13)	8 (35)	6 (26)	19 (83)	2 (9)	13 (57)
	**R**	6 (26)	7 (30)	9 (39)	8 (34.8)	3 (13)	20 (87)	15 (65)	17 (74)	4 (17)	21 (91)	10 (43)
*K. pneumonia* (n = 14)	**S**	5 (36)	2 (14.3)	2 (14.3)	7 (50)	11 (79)	2 (14.3)	4 (29)	6 (43)	9 (64.3)	-	8 (57.1)
	**R**	9 (64)	12 (85.7)	12 (85.7)	7 (50)	3 (21)	12 (85.7)	10 (71)	8 (57)	5 (35.7)	14 (100)	6 (42.9)
*P. aeruginosa* (n = 11)	**S**	9 (82)	2 (18.2)	3 (27.3)	-	11 (100)	Nt	4 (36.4)	2 (18.2)	11 (100)	-	-
	**R**	2 (18)	9 (82)	8 (73)	11 (100)	-		7 (63.6)	9 (82)	-	11 (100)	11 (100)
**Total (n = 77)**	**S**	**45 (58.4)**	**30 (39)**	**32 (42)**	**39 (51)**	**58 (75.3)**	**5 (7.6)**	**27 (35.1)**	**20 (26)**	**58 (75.3)**	**3 (4)**	**37 (48.1)**
	**R**	**32 (41.6)**	**47 (61)**	**45 (58)**	**38 (49)**	**19 (24.7)**	**61 (92.4)**	**50 (64.9)**	**57 (74)**	**19 (24.7)**	**74 (96)**	**40 (51.9)**

KEY: S = Sensitive R = Resistant; −: zero; Nt: Not tested; CN: Gentamicin; C: Chloramphenicol; SXT: Trimethoprim-sulphamethoxazole; NOR: Norfloxacin; KF: Cephalothin; CRO: ceftriaxone; TE: Tetracycline; CIP: Ciprofloxacin AP: Ampicillin; DO: Doxycycline.

### Multi- drug resistance patterns of the isolates

Multi-drug resistance (MDR) test was determined by disk diffusion method according to criteria set by CLSI 2011 against different class of antimicrobials: penicillin class (penicillin G and ampicillin), cephalosporin class (ceftriazone and cephalothin), glycopeptides class (vancomycin), aminoglycopeptides class (gentamicine and amikacin), Macrolides class (erythromycin), tetracycline class (tetracycline and doxycycline), fluoroquinolones class (ciprofloxacin and norfloxacin), folate pathway inhibitors (sulphamethozaxole trimthoprim) and phenicols class (chloramphenicols) for gram positive bacteria and penicillin class (ampicillin), cephalosporin class (ceftriazone and cephalothin), aminoglycosides class (gentamicin), tetracycline class (tetracycline and doxycycline), fluoroquinolones class (ciprofloxacin and norfloxacin), quinolones class (naldixic acid), folate pathway inhibitors (sulphamethozaxole trimthoprim) and phenicols class (chloramphenicols) for gram negative bacterial isolates.

Multi- drug resistance was found in 123 (85%) of the isolates. Seventy one percent (71%) of the gram positive bacterial isolates showed multi drug resistance (two – nine antimicrobial classes). Similarly, 97.4% of gram negative bacterial isolates showed MDR (against two to eight) antimicrobial classes. Furthermore, 100% of *P. aeruginosa* was resistant to two or more than two antimicrobial classes (Table 5).

**Table 5:** Antibiogram of bacteria isolated from patients with infected wounds at JUSH, Jimma, May-September, 2013

**Antibiogram**							
**No. (%) of resistance**							
**Organism**	**R2**	**R3**	**R4**	**R5**	**R6**	**R7**	**R8**
**Gram positive**	15 (32)	8 (17)	4 (8.5)	1 (2.1)	2 (4.3)	1 (2.1)	0
*S.aures *(n = 47)							
CONS (n = 21)	5 (24)	3 (14.3)	4 (19)	1 (4.8)	0	3 (14.3)	1 (4.8)
**Total (n = 68)**	**20 (29.4)**	**11 (16.2)**	**8 (12)**	**2 (3)**	**2 (3)**	**4 (6)**	**1 (1.5)**
**Gram negative**	2 (7)	5 (17.2)	4 (14)	3 (10.3)	2 (7)	8 (28)	5 (17.2)
*E.coli* (n = 29)							
*Proteus Spp* (n = 23)	6 (26.1)	5 (22)	3 (13)	0	3 (13)	4 (17.4)	1 (4.3)
*K.pneumoniae* (n = 14)	1 (7.1)	0	0	3 (21.4)	4 (29)	2 (14.3)	3 (21.4)
*P.aeruginosa* (n = 11)	1 (9.1)	0	2 (18.2)	2 (18.2)	3 (27.3)	3 (27.3)	0
**Total (n = 77)**	**10 (13)**	**10 (13)**	**9 (12)**	**8 (10.4)**	**12 (16)**	**17 (22.1)**	**9 (12)**

R2-R8 = number of antimicrobial class in which a given isolate was resistant.

### Minimum inhibitory and bactericidal concentration of alternative topical agents

In vitro susceptibility of the isolated organisms to alternative topical antimicrobial agents was studied. All of the tested microorganisms were sensitive to acetic acid at a concentration of 0.5% (V/V) and this concentration is bactericidal when applied for 10 minute to all clinical isolates in the study. All of the isolated organisms were resistant to a concentration of 0.025% (V/V), 0.25% (V/V) of dabkin solution after incubation for 24 hrs. However, the solution was bactericidal at a concentration of 0.5% (V/V) for 58.6% of all the isolates while 100% of the isolates were killed at 1% (V/V) of the solution (Table 6). These concentrations were lethal to the isolates if applied for 10 minutes. The minimum inhibitory and bactericidal concentration of the solution (dabkin solution) was 0.5% and 1%. The other topical agent frequently used in the study setting was 3% Hydrogen peroxide. This solution was bactericidal to all isolated organisms from infected wound. In vitro test indicated that this solution was not bactericidal when applied for less than 10 minute.

**Table 6:** Minimum inhibitory concentrations of alternative topical agents against bacterial isolates from patients, JUSH, May-September 2013

**Antimicrobial agents**	**Isolates**	**0.025% (V/V)**	**0.25% (V/V)**	**0.5% (V/V)**	**1% (V/V)**
Dabkin solution	*S. aureus*	All isolates were resistant	All isolates were resistant	35 (74.5%)	47 (100%)
	CONS			15 (71.4%)	21 (100%
	*E.coli*			8 (28%)	29 (100%)
	*Proteus Spp*			15 (65.2%)	23 (100%)
	*K.pneumoniae*			10 (71.4%)	14 (100%)
	*P.aeruginosa*			2 (18.2%)	11 (100%)
	**Total**			**85 (59%)**	**145 (100%)**
Acetic acid	**0.5% (V/V)**				
	*S. aureus*	47 (100%)			
	CONS	21 (100%)			
	*E.coli*	29 (100%)			
	*Proteus Spp*	23 (100%)			
	*K.pneumoniae*	14 (100%)			
	*P.aeruginosa*	11 (100%)			
Hydrogen peroxide	**3% (W/V)**				
	*S. aureus*	47 (100%)			
	CONS	21 (100%)			
	*E.coli*	29 (100%)			
	*Proteus Spp*	23 (100%)			
	*K.pneumoniae*	14 (100%)			
	*P.aeruginosa*	11 (100%)			

## Discussion

The incidence of wound infection was more common in males (89.7%) than in females (81.4%). This is in agreement with studies done in different parts of Ethiopia [7-10] and other countries [11-13]. This might be explained by the fact that traditionally, in this country mainly males are involved in occupations such as farming, construction works, transportation and industry works where the likely exposure to trauma is common.

In this study, 91.6% of culture positive wounds showed mono-microbial growth, 8.4% showed poly-microbial growth and 12.7% had no bacterial growth. Similarly high percentage of mono-microbial growth was reported in India (86-100%) and Pakistan (98%) [14-17].

In our study, *S. aureus* (32.4%) and *E. coli* (20%) were the predominant organisms isolated from wound infections. A number of reports done previously on wound infection from Ethiopia and different parts of the world indicated that *S. aureus* and *E. coli* were the most frequent isolates [18-21]. The high prevalence of *S. aureus* infection may be because it is an endogenous source of infection. Infection with this organism may also be due to contamination from the environment e.g. contamination of surgical instruments. With the disruption of natural skin barrier *S.aureus*, which is a common bacterium on surfaces, easily find their way into wounds.

Coagulase Negative *Staphylococci (CONS)* accounted for 14.5% of the organisms isolated from wounds in this study. This is not unexpected since the organism is a commensal or normal flora on the skin. Several investigations have reported these organisms as common contaminants of wounds [9,20].

Resistance to the selected antimicrobials was very high. The average resistance of the isolates to all the antibiotics in gram positive cocci was (99%) and gram negative bacilli (100%). This is similar to the study done in Ethiopia with average resistance of gram positive cocci isolates (100%) and gram negative bacilli isolates (95.5%) respectively [20]. The overall multiple drug resistance (two and above antimicrobial classes) of the isolates in this study was 85% which was in line with previous study done in different parts of the world [8,19,22]. High resistance of the isolates to antibiotics may be due to practicing self medication, lack of diagnostic laboratory services or unavailability of guideline regarding the selection of drugs thereby which lead to inappropriate use of antibiotics.

In the determination of the susceptibility of *S. aureus* on fifteen selected antibiotics by disk diffusion technique showed that *S. aureus* tend to be resistant to a wider spectrum of antibiotics. In this studies *S.aureus* was highly resistance to ampicillin (95.7%), penicillin (91.5%) and tetracycline (51%). This was consistent with study done in Ethiopia [19,20] and elsewhere [4,11,14,23]. The same isolate was highly sensitive to amikacin (100%), vancomycin (100%), ciprofloxacin (96%), norfloxacin (96%) and gentamicin (96%). This finding is in agreement with the work of Bess LJ. *et al.*, Bibi S. *et al.*, Shamsuzzaman *et al.*, Gelaw A. *et al.*, Gautam R *et al.*, and Shriyan A. *et al.,*[7,15,23-26], who reported that clinical *Staphylococci* are 100% sensitive to vancomycin and [24,27,28] to amikacin. In this study, coagulase negative *Staphylococci* were 100% sensitive to amikacin and vancomycin, sulphamethoxazole trimethoprim (86%), gentamicin (83%) and ciprofloxacin (76.2%). This finding was comparable with the previous studies done in different parts of the world [11,28]. The same organism was remarkably resistance to ampicillin (90.5%), penicillin (76%), cephalothin (71%) and tetracycline (52%). This finding was comparable with study done in the same country [20,29,30] and in other parts of the world [4,11,14,31]. Remarkable susceptibility of gram positive bacteria to vancomycin, amikacin and aminoglycosides (gentamicin) may be due to lesser use of these antibiotics as a result of their less availability, cost and toxic effect respectively.

In this study, 100% of the *E.coli* isolates were resistant to cephalothin, ampicillin (96.6%), tetracycline (79%), chloramphenicol (65.5%), ceftriaxone (62%), sulphamethoxazole trimethoprim (55%) and gentamicin (51.7%). Sensitivity pattern of *E.coli* in our study as compared to others were ciprofloxacin (65.5%) and naldixic acid (59%) [8,19,28,29]. So, reduced antibiotic sensitivity pattern noted for *E. coli* suggests its importance for hospital acquired infection.

*K. pneumoniae* was 100% resistance to ampicillin, 85.7% in chloramphenicol, sulphamethoxazole trimethoprim and cephalothin, (71%) in ceftriaxone however it indicates low resistance to ciprofloxacin (35.7%) and doxycycline. This was in consistence with the study done in Ethiopia [7,8,20,30]. *Proteus* species were resistance to ampicillin (91%), cephalothin (87%), tetracycline (73.9%) and ceftriaxone (65%). The isolates were sensitive to ciprofloxacin (83%) and gentamicin (74%). This was compared to previous studies done in the country [8,29,30] and elsewhere [25,32,33]. Most of the gram negative bacteria isolated were resistant to ampicillin, cephalothin, tetracycline and chloramphenicol. This may be due to the antibiotics having been in use for much longer time and their oral route of administration that affects their rate of absorption into blood stream. Some of them were used as prophylaxis therefore increasing their use in patients. Over use of antibiotics contributes to organisms developing resistance.

In our study *P. aeruginosa* showed reduced sensitivity to commonly used antibiotics like ampicillin, doxycycline, naldixic acid, and tetracycline, except ciprofloxacin, norfloxacin (100%), and gentamicin (82%). Ciprofloxacin and norfloxacin has been stated to be the most potent oral drug available for the treatment of *P. aeruginosa* infections. This report is in conformity with the result of other study in which ciprofloxacin recorded the least resistance (6.2-24%) to *P. aeruginosa* isolates from wound infection [20,29,34]. It is undoubtable that at the present time, the oral drug ciprofloxacin and injection gentamicin are the most effective antibiotics against *P. aeruginosa* involved in wound infection relative to most other commonly used drugs. *Pseudomonas* resistant to third generation cephalosporins (ceftriaxone 63.6%) is real treat. In fact, the irrational and inappropriate use of antibiotics is responsible for the development of resistance of *Pseudomonas* to antibiotic monotherapy. The incidence of *P. aeruginosa* in wound infection among admitted patient is becoming more serious in developing countries because of lack of general hygienic conditions, production of low quality antiseptics and medicinal solutions for treatment [11].

The use of acetic acid has been reported from time to time as a topical agent for the treatment of *Pseudomonal* infections and in most reports has been used for burns and superficial infection. Topical use of acetic acid at concentrations between 0.5 to 5% eliminated *P. aeruginosa* from the burns and soft tissue wounds of 14 out of the 16 patients within two weeks treatment [35]. Even though, the former study was in vivo the same result was obtained in vitro in this study in which 0.5% acetic acid was bactericidal for *P. aeruginosa*.

Acetic acid had high bactericidal effect than dabkin solution tested at 0.5% concentration against clinical isolates obtained from infected wound. Acetic acid was bactericidal at 0.5% concentration, which can be used clinically because it was highly diluted and non toxic. Other study done on acetic acid antimicrobial effect and toxic effect indicate that this concentration of acetic acid was non toxic, easily available [36]. Furthermore in this study 0.5% acetic acid was bactericidal for gram positive (*S.aureus* and CONS) and gram negative bacilli (*E. coli, K. pneumoniae, Proteus* spp and *P. aeruginosa*). Majority of the study done so far used this topical agent for the treatment of *P.aeruginosa* in the concentration interval of 0.5-5% [35].

Study have been done on sodium hypochlorite solutions to determine its bactericidal and wound-healing properties with concentrations of 0.25%, 0.025%, and 0.0125% at 5-, 10-, 15-, and 30-minute intervals indicated that bactericidal effects were observed for concentrations as low as 0.025%. While, tissue toxicity, both in vitro and in vivo, was observed at concentrations of 0.25% but not at a concentration of 0.025% [37]. However in this study the solution was not bactericidal at concentration of 0.025% and 0.25%, this may be due to increased resistance of bacteria to the solution; this solution was bactericidal in our setting at the concentration of 0.5% and 1%.

Antimicrobial activity and effectiveness of a combination of sodium hypochlorite and hydrogen peroxide in killing and removing *P. aeruginosa* biofilms from surfaces showed either a significant reduction or complete removal of biofilm material after a 5 min exposure to the mixed sodium hypochlorite and hydrogen peroxide solution [38]. Hydrogen peroxide in the current study setting was bactericidal to all isolates when exposed to the isolates for the minimum of 10 minutes otherwise 20% of the total isolates were resistance when applied for less than 10 minutes.

## Conclusion

The most common isolate in wound infection was *S. aureus* followed by *E. coli, Proteus* species*, CONS and K. Pneumoniae.* These isolates showed high frequency of resistance to ampicillin, penicillin, cephalothin and tetracycline. Seventy one percent (71%) and 97.4% of Gram positive and Gram negative isolates showed MDR respectively with overall MDR of 85%. On in vitro sensitivity testing, acetic acid at a concentration of 0.5% (V/V), dabkin solution at concentration of 1% (V/V) and 3% hydrogen peroxide were bactericidal against all clinical isolates.

## Competing interests

The authors declare that they have no competing interests.

## Authors’ contributions

MM, TS and AA participated in design, laboratory analysis, interpretation of the data and write up of the manuscript. All the authors read and approved the final version.
